# Chronic Ischemic Heart Disease Affects Health Related Quality of Life

**DOI:** 10.4021/cr236w

**Published:** 2012-11-20

**Authors:** Abolfazl Goreishi, Zahra Shajari, Zeinab Mohammadi

**Affiliations:** a Metabolic Disease Research Center, Zanjan University of Medical Science, Iran; b Department of Psychiatry, Zanjan University of Medical Science, Iran

**Keywords:** Quality of life, Chronic congestive heart disease, Depression, Anxiety

## Abstract

**Background:**

Chronic diseases endanger not only physical health but also psychological and social health of patient. Thus, evaluation of such patients for psychological treatment decisions is very important.

**Method:**

This is a descriptive study that was performed with 50 chronic patients (ischemic heart disease) selected from Valiasr and Mousavi at cardiac wards in Zanjan Province. They were given three types of questionnaire: demographic, WHOQOL, and Zung depression and anxiety index. The information was statically analyzed by frequency chart, central indexes, dispersion, Chi-Square and t tests, Pearson’s correlation index (P < 0.05).

**Results:**

The average of quality of life in all patients were calculated as was respectively 12.19, 11.98, 12.08, and 12.4 in physical, psychological, social and environmental domains respectively, 68 percent of total number of the patients had various degrees of anxiety and 78 percent of them had various degrees of depression. There was a significant relationship between the life quality average in all domains and anxiety intensity and depression intensity (P < 0.05) and there was a significant relationship between life quality average in all domains and income (P < 0.05).

**Conclusion:**

As the level of depression and anxiety goes up, quality of life decreases pointing out that they have a reverse relationship. Depression and anxiety are one of the most significant factors of quality of life among other variables. Regarding specific conditions of the treatment, it is necessary to pay special attention to psychological aspects.

## Introduction

Chronic diseases endanger not only physical health but also psychological and social health of patient seriously and many of them live without hope to improve. Generally, cardiac disease is among such chronic diseases. Chronic ischemic heart disease (IHD) is common with rising age [1].

Congestive report of disease management published in Iran in 2001, the number of patients with heart disease in 18 provinces of Iran counted around 3,337 cases per 100,000. The 1998 survey showed that 25% of patients’ admissions in cardiac wards were related with chronic heart disease [2].

Goals of treatment in ischemic heart disease are to modulate symptoms and improve the prognosis. Chronic heart disease is associated with severe limitations of quality of life (QOL) [3, 4]. QOL constitutes the individual’s perceptional symptoms, well-being, and physical and mental functional capacity. Over the past decade, quality of life improvement has been increasingly used as an indicator of health outcome [5, 6]. So, another major goal of health care is to maximize function in everyday life and to elevate level of quality of life. Quality of life is supposed to have a relatively new scientific role to evaluate effectiveness of treatment strategies and the course of a disease [7-9].

In this context, we assess the influence of socio-demographic variables, objective measures of age, sex, heart disease severity and duration, anxiety and depressive co-morbidity on the quality of life in a cross-sectional analysis in patients with CHF. Thus, we had a particular interest to evaluate the quality of life in chronic heart diseases and affecting factors. Additionally, it is unknown whether there is relationship between depression and anxiety with QOL similar across different chronic conditions; we tried to clarify these aspects.

## Material and Methods

A descriptive-cross sectional study was designed to evaluate quality of life and mental health condition of chronic heart disease patients selected from congestive heart disease care unites and wards of Zanjan hospitals; Vali-e-asr and Mousavi. This study was approved and supported in the Zanjan University of medical sciences in 2011.

### Study population and design

A total of 50 chronic heart disease volunteers were selected randomly from the patients who referred to congestive heart disease wards of vali-e-asr and Mousavi as referral academic hospitals, in Zanjan. The inclusion criteria of eligible patients enrolled in this study was aged of 30 - 80 years, with chronic ischemic heart disease dysfunction at least for 6 months. Subjects were excluded from the study if they were under 30 or over 80 years old, or if they suffered from new onset of congestive heart disease, or other co-morbidity such other simultaneous chronic somatic of psychological disease. Subjects gave their informed consent to participate in the present study. All subjects were asked to complete their demographic and medical information including age, sex, education, disease duration and its severity, substance abuse history, psychological disorder history in a questionnaire. Then, patients completed 26 question containing WHOQOL-BREF questionnaire which assessed 24 factors in quality of life in 4 parameters; physical, psychological, social and environmental. Also, the patients were asked to answer some self-rating questionnaires: the Zung Depression [10, 11]. All processes were conducted under supervision of general physician and the details of the study were explained to the entire participant. Questionnaire of illiterate participants were completed by her.

### Health-related quality of life assessment

The ischemic heart disease was defined a clinical syndrome because of an inherited or acquired abnormality of congestive heart disease structure and/or function, develop a constellation of clinical symptoms (dyspnea and fatigue) and signs (edema and rales) that lead to frequent hospitalizations, a poor quality of life, and a shortened life expectance [12].Consecutive patients with stable ischemic heart disease were selected according to the NYHA functional class by assessment of electrocardiography, exercise test, echocardiography or angiography documents.

### Quality life assessment

The World Health Organization Quality of Life-BREF (WHOQOL-BREF) is a 26 containing items designed by World Health Organization (WHO) to measure 24 factors affecting quality of life in 4 components representing physical, psychological, social and environmental functioning. Also, 2 question assessed total quality of life and general health condition. Scale scores are then transformed to a 0 to 20 range. The lower score the more poor quality life condition. Validity and reliability of WHOQOL-BREF questionnaire was determined first time by Dr Nejat and colleagues, 1,167 subject from Tehran completed this questionnaire and then its reliability was confirmed by Cronbach score (Cronbach alpha = 0.83). Intra class correlation and Cronbach alpha score in all domains were 7/0, respectively, but in the social domain alpha was calculated 55/0, which can be due to low number of questions or inquiries in this area. Questionnaire validity of this tool with the ability to distinguish healthy and patient groups were evaluated using linear regression and correlation matrix to assess the structural factors of the questionnaire were used. In 83% of cases, the correlation of each question with its main areas was higher than other areas. Results obtained in this study showed acceptable validity and reliability, and structural factors of this tool in healthy and patient groups, in Iran [13].

### Statistical analysis

All descriptive statistics are presented as means and standard deviations for continuous variables, and as absolute and relative frequencies for categorical variables. Health related quality of life scores are presented as means and standard deviations.

Comparisons were performed with 2-sample t tests (for continuous variables) and chi-square tests (for categorical variables). Data were evaluated by soft ware SPSS version 16. Significant P-value was considered as P < 0.05.

## Results

### Characteristics of chronic ischemic heart disease subjects

A total of 50 patients fulfilled inclusion criteria and participated in the study. Their characteristics are summarized in Table 1.They were in a mean age of 58.6 years old, 60% female. More than 85% suffered from moderate and server ischemic heart failure (according to York Heart Association (NYHA) functional class) but less than 120 months, 60% were illiterate and 80% had economic income less than 300$ monthly. Psychological problem including anxiety or depression was found in only 16% of subjects which was not statistically meaningful.

**Table 1:** Baseline Characteristic of Congestive Heart Failure Patients

Variables	Number (%) N = 50	P value
Sex		
Male	20 (40)	P > 0.05
Females	30 (60)	
Education level		
Illiterate	30 (60)	P > 0.05
Primary school	13 (24)	
High school/university	7 (16)	
Marital statute		
Single	0	P > 0.05
Married	47 (94)	
Divorced	1 (2)	
Worth	2 (4)	
Duration of congestive heart failure		
6 - 12 months	21 (42)	P > 0.05
12 - 120 months	21 (42)	
< 120 months	8 (16)	
Income		
< 300$	40 (80)	P > 0.05
> 300$	10 (20)	
Insurance of health care(NHS)		
Yes	43 (86)	P > 0.05
No	7 (14)	
Drug abuse history		
Yes	7 (14)	P > 0.05
No	43 (86)	
Psychological problem		
Yes	8 (16)	P > 0.05
No	43 (86)	
Anxiety severity		
Normal	16 (38)	-
Mild/moderate	22 (44)	
Sever	12 (24)	
Depression severity		
Normal	11 (22)	-
Mild/moderate	29 (58)	
Sever	10 (20)	

### Quality of life score

Scores in each parameter in physical, psychological, social and environmental domains evaluated in WHOQOL-BREF questionnaire was respectively 12.19, 11.98, 12.08, and 12.4. It was revealed all parameters of quality of life had a significant correlation with sex and educational level and income (P < 0.05). It means that educated women with high income experienced better mental health and quality life. Age, duration of disease, substance abuse history, and Psychological problem history were not correlated with quality of life components.

### Anxiety and depression values

Severity of anxiety and depression among patients was shown in Table 1. Approximately one fifth of patients expressed severe symptoms of anxiety and one forth suffered from severe depressed mood. Evaluation of quality of life parameters relationship with anxiety and depression showed a statistically meaningful correlation in those ischemic heart disease patients. In congestive heart disease, quality of life decreases as anxiety and depression severity worsens (P < 0.05) Table 2.

**Table 2:** Relationship Between Quality of Life Score and the Degree of Anxiety in Congestive Heart Patients

Health domain of quality of life	Degree of anxiety	Number	Quality of life score	SD	P value
Physical	Normal	16	13.2	1.5	0.001^1^
	Mild/Moderate	22	12.3	1.4	
	Sever	12	10.5	2.6	
	Total	50	12.1	2	
Psychological	Normal	16	13	2.2	0.01^1^
	Mild/Moderate	22	11.9	1.9	
	Sever	12	10.6	1.9	
	Total	50	11.9	2.2	
Social	Normal	16	13.7	3.3	0.02^1^
	Mild/Moderate	22	12.7	3.5	
	Sever	12	10.1	3.2	
	Total	50	12.4	3.6	
Environmental	Normal	16	14.3	3.3	0.003^1^
	Mild/Moderate	22	12.3	2.3	
	Sever	12	10.1	3.6	
	Total	50	12.4	3.3	

^1^P value < 0.05 is considered statistically significant.

**Table 3:** Relationship Between Quality of Life Score and the Degree of Depression in Congestive Heart Patients

Health domain of quality of life	Degree of anxiety	Number	Quality of life score	SD	P value
Physical	Normal	11	13.3	1.8	03/0^1^
	Mild	19	12.6	1.7	
	Moderate	10	11.9	1.3	
	Sever	10	10.3	2.4	
	Total	50	12.1	2	
Psychological	Normal	11	14	1.6	< 0.05^1^
	Mild	19	12.3	1.9	
	Moderate	10	11.1	1.7	
	Sever	10	9.8	1.5	
	Total	50	11.9	2.2	
Social	Normal	11	15.4	2.8	< 0.05^1^
	Mild	19	13.3	2.9	
	Moderate	10	10.7	3.7	
	Sever	10	9.3	2.4	
	Total	50	12.4	3.6	
Environmental	Normal	11	15.4	2.6	< 0.05^1^
	Mild	19	13	2.6	
	Moderate	10	11.2	3	
	Sever	10	9.4	2.4	
	Total	50	12.4	3.3	

^1^P value < 0.05 is considered statistically significant.

## Discussion

Chronic heart disease (CHF) is associated with impaired quality of life (QOL) due to repeated hospital admissions, and a bad prognosis. Many patients suffer from depressive and anxiety symptoms with additional negative impact on prognosis [1, 3, 4, 14-17].

In this cross sectional study, we evaluated the impact of socio demographic variables, objective measures of heart disease duration, anxiety, and depressive co-morbidity on the QOL in patients with CHF treated in hospital cardiac wards.

We found that socio demographic factors such as age, duration of disease, substance abuse history, and psychological problem history weakly correlated with some QOL scales. But aspect of quality of life may improve in female sex, high education level and income.

Inconsistent previous findings showed worse QOL in women than men, others found that association only in the physical domains of QOL10 or not at all [18, 19]. Several reasons for this inconsistency have been discussed, such as different symptom perception or depression rates in women in comparison in men [3, 19]. Our patient sample was predominantly female, therefore our sample had only 40% male, which was potentially the reason why sex had only a marginal impact on QOL [20].

Similar to our study with a population of 184 showed that approximately 50% of the patients’ quality of life is undesirable, type of job, income overall life education, home care, disease severity, disease duration, frequency of doctor and hospital admission, duration of hypertension, edema, hyperlipidemia, fatigue and various medications were associated with significantly with quality of life scores [2].

Chronic heart disease duration played no or only a minor role in predicting QOL. But significant correlation between heart disease duration and QOL has been described in some studies which evaluated acute unstable cardiac angina admitted in cardiac care units [21, 22].

The relationship between depression and quality of life (QOL) has been studied in many chronic condition including populations with ischemic heart disease (IHD). The prevalence of depression in this group seems to be higher than in the general population, ranging from 15 to 50% [22-25].

Similar to previous findings, our results show that patients with chronic HD experiences different signs of depression, anxiety, and emotional stress [26, 27]. Data from Brazil study revealed that depression and anxiety prevalence was considerable and depression is an independent factor associated with worse QOL in congestive heart disease. Among the priorities aiming at improving QOL must be evaluation and management of depressive symptoms [28].

In the recent study, anxiety and depression had a statistically meaningful correlation with quality of life in those congestive heart disease patients and quality of life decreases as anxiety and depression severity worsens. These findings are consistent with data reported by other authors considering depression and anxiety as the variable most predictive of worse quality of life [24, 29, 30].

### Conclusion

Our study reinforces the more severe depression and anxiety experience, the worse quality of life is supposed in chronic illness. Our findings also highlight the importance of assessment and management of depression and anxiety in the IHD. It can be deduced to pay special attention to psychological aspects.
